# Supplementary material for: Uncovering the essential roles of glutamate carboxypeptidase 2 orthologs in Caenorhabditis elegans
Source: Biosci Rep. 2024 Jan 12;44(1):BSR20230502. doi: 10.1042/BSR20230502 (PMC10794815; doi:10.1042/BSR20230502)
Supplement: Supplementary Figures S1-S10 and Tables S1-S6 [file BSR-2023-0502_supp.pdf]

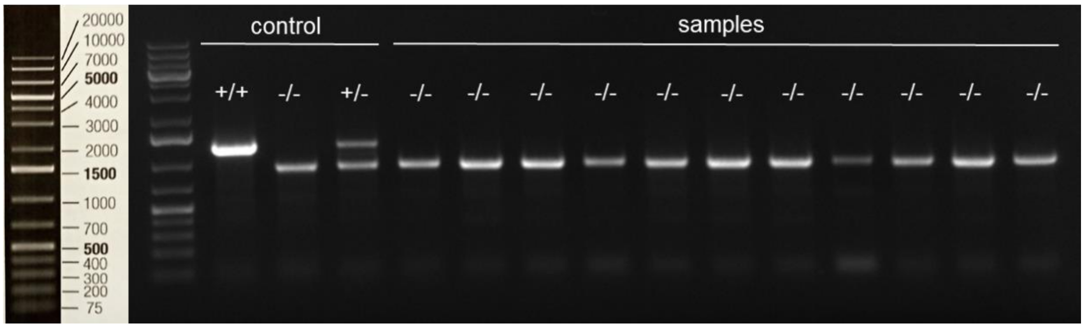

Figure S1. Gel after the backcrossing (*gcp-2.3* KO example). The indication in the figure is +/+ for wild type, +/- for heterozygotes, and -/- for homozygous mutants.

Caenorhabditis elegans, gcp-2.1 promoter area (2898 bp)

ttttcaggaggacccttggaggctcaaccacttcatttatttatctttttttaagttcacc
cttcagcataccaaaggttacattttttgcagcagtgcacttataatgttgataataataa
aatattaattggaatatgatgtttgttatcttatcaggcaatagtcgacattgataaaaa
ttggaagattgctttcaaattgatttttttttccaattattttactttttgaaacatttc
attttacaattctattgttccttgagaaaaaaacgtcataagttttgatttttttgcaatg
cgttcagttatttctgaacgaaacgtatttcatttgaaaggacttcttctcaatagtcc
actacaaataatttttatgcatattcaggtcaaatgtttattttatgaagtttcatagac
atggagacattttttatatctttcaagacagcctttcaaaaaattagtgaaattgtttaat
caagtccattaaaaatgttaaataagtaaaatgtttgtcgaatttttttgaatgttttttt
tgagttactggagaatgaatcaaatgatttaaagtggtcgtttttttacagtaaaaaagta
ttatattttgaatgactaatgtatttatttgataatataatttctttcagtaagatactt
tatgtgtcctatgcctttccacttctaaagccagaagtgaactgattggaaattttttttt
taagtttcaattttgtacccaaccgtagagttcgtatatacgagcattctcattaatttg
ccagtgtttcaaaacaaaaattccaagaaaaaaaaactctataattgcctcatttcttct
gttagtctggctgcttgaattttgaattataaatgttgactcaggacttattgccagccc
taattaaaaagagtattttttctattagcatggtatttgtattgcacacatttttttacac
aattcctatgagattgaagttattaagcctttttgttacaattaaaataaaattgtcgaa
cttccatataaagtttaagtcttttcaagtaaaaaaatagtaagtccaaacacagaaaaa
tgttctgaaaattccctaataacggaatgacctttgaaaattagtattgtagccatacta
acaaaagaaatacggcaataatatcattataaaatattgtagtatagctataacgcaaat
accaaaccttcatcagtgtgtgtaatagaaagaacaagtgtcctactcaaacataccaat
aaacatacaacaataaggtgggcccagggagcgcacgtatcatatcctctttttctacac
atacaccagatgatggagcatcatcagacaactgacttcaattcgatgacactctggatt
atatgtatcaagagggggaaacaattgaggggagggggcagatgaaagaatcgtcgcttg
tgattctcaattcaattttcatttttctgtgtctgtttacaatattagtagagaaaagca
tttgatatatgaactaatgttttggggtttgtatcatagtttttttaatcacaaaactgaa
aactttattttttatatattgttcatgcaaaaatgttagcatttccggttcatgatgtttt
ctatcaattttttgttgtgattacatttgcaaaattatttatttaatcttaaaggtggagt
agtgccagtgggaattttgtctaaatacacttattttgatcaaaaacgatcgaataccat
aatataacgttccaaaaattttttttagtgttttcataatttctgggcgaagttttggca
aattgccataattttgaaaaataagagcttttgaggaaatccaaagaaattacgcttatt
ccgagccctatgtacaatgtttcaatacaaataattaaaacaaaattgcactataaaaaa
taaaggaaaattttttttttggtcgactttcaaaatgagtggcaaaaacgaagtaattgt
cacttttttgacagttgataaaaaaatgttcaaaaacctttttgaaaagttttattgtgatct
ttggtcattttgggaccaaatgagtggtttataacaatttccccacttgcgctactccac
ctttaaaacaccaaaaagtgttaggctgttctatataatttgtgcccaaaaaatatgaca
tcagcatgttcttaacaatgaaaaatctgttgagaactctgagtctcttctcctgcattt
tttcaatagatctacgtagatcaaaccgaaatttttttcgtgcatttttttcagtcttag
aagggatagtttacttggcctaagaatacaaaaaatccacaattttgtttctataatcga
agaagtattgttttaaaatgcatgtatgtgatacaaatgaattaaattttaaacaaaatt
gaaaaattactgttttgaaacctgcaaatcttatctggcctaaatcaatgttgacagtcc
gtttacaatgagtttcattttttgttgctcagatatcaaatacacaattacacgtaaactt
ttgaaaagtgtattcatagtttcaaagttttcgtttttttcttcgtccagtcatttcttac
agtaattttttgattaagttcttaactgtaactgtaattaattcaactccacttttaaata
atctcgcaacatttgtttgaaatgttgttgttccagaacaatagtttatatgtaaatgcc
gtacgtcggagttggagcacaaacagtttccacaagcttaacaggagcgcctatggtgaa
ggcatacattgcaattgctgcctctttgatttttgtttttttgtattgctgcgttgggtgt
tcatcactcggaaagaaagttcaacaaattcaataaagtttcaattgatgacattcataa
atctgatgcagga

Caenorhabditis elegans, gcp-2.2 promoter area (1107 bp)

ttttcaggaggacccttggaggagagataattttcatttctatgattgtccgtttttgtt
cgttatgcataatgttttgactcctttcattacagtttgacacatttgtaaacgtttata
atcttcaataaacaacataagattttttttttgtaaaacctaacttataatctgacattt
tacgcttaatcaggttttttttatttacggtaaatagaaagttttttcaacaaactaaac
ttgaaggggtttttttttgcatcataagaaatggttctatacttcatcttggactttgat
atgaaatagtaatttttcgattaaagagtgttactgttagtaactgcaaccgctttcgtt
gcagcagagaaggttcagccatgaatgatgttttgaaatatcaaaactaaattcaatggg
aatccatgatctcaccaaataataacactctgaaccttccagtttgattatttttcagta
ttctataaatacttttttttatttcatttcaaacattcagtcgaataattgagcctgatt
ttcaaatgatccattatccccgagtttaagcagttttgagttgttaaagtcttgattatt
aatcaataaaaacaaagttgtattcgatctttgtttaatgtatctttcgtgctcagattt
tattaggacatatcatatatgagtttttatatttgaaaaaaaccaatcagtcacacacaa
ttttccatcgtcgtttatacttttatgggcgttgaaacgacaacccacattttcccaaaa
aattttcttccttgactactctgaatatatttctattaaattcattttgtaaaatttga
tcctaatttaagtgtagaaaatgataacagctcggaaccggtttacacaaggttgtagtt
gtatgaagtattataacaataatggagacaaagtattccaaaagtctaaaagaaatagag
gaatgaaagctagcggtgtggttcttgttgcagtttctactgttgctttgactattattt
tatctaatgcgatacatcaatcttacaaatcaaattcaaagcctcttcctaaattatcaa
ttgtaccggtagaaaaaatgagtaaag

Caenorhabditis elegans, gcp-2.3 promoter area (911 bp)

ttttcaggaggacccttggaggtcacgtttgatagactctcagagtaataattatttaaa
tgtcaactttttaaagtgtaaggtggagtagtgcaagtggggaaagtgtttaaatggtga
aaatgaccaaaaataattgcaaaacattacaacaaattttggaatgtttttatttactg
caaaaaaatgatacctactcagtttttgccagtgcgacataagtctggatttgcttgaaa
gcttacagttacagaatttttttaaaaactcttgaattttggagtgttttattgcgatat
tcagacaaaatccccactggcgatgtttactatttaaccaataaatgtattttgaataca
gtaacgttgagaaacttgacattaaaaaactcacagaaaatttcgaattagttttca
tttgacccgttcgaatgtacattcgttcaaatttcaaccaacacattccaacggaagttt
gatgcctgttcaaaaatcatggacagattatttttttaatcaggaacgagagcatgtcga
acacatctaacgccgaaaactagtttttatcaggaacttctgaattaaatccctaaaata
atgtattcttacgaataaataatgaaactatttttttaaactcgtcgactttgccagtaat
atttgcattttatcaaagcatataaatataacctggtaaacgaatcacttttctatgata
taacgtttttcaaaaatcgaacatgaaaaaaggattacaaatattcggcgttgtattact
tctagcagcgacagttgttgtaactgtactcatttcaaattatgtacatcagctttcaat
gtcgagtgggacacctacaatacaaaatacagtttcaatcgcaaatgtaccggtagaaaa
aatgagtaaag

Figure S2. Promoter region for _gcp-2.1_, _gcp-2.2_ and _gcp-2.3_.

```
CeGCP2.1a      MPYVGVGAQTVSTSLTGAPMVKAYIAIAASLIFVFCIAALGVHHSERKFNKFNKVSIDDI 60
CeGCP2.1b      -------------------MVKAYIAIAASLIFVFCIAALGVHHSERKFNKFNKVSIDDI 41
CeGCP2.1c      -------------------MVKAYIAIAASLIFVFCIAALGVHHSERKFNKFNKVSIDDI 41
                                  *****************************************

CeGCP2.1a      HKSDAGVIQDNIKTENIKKYLRIFTKDPHVAGTEANKKVAYEIANAWSEAGLEDVHTLPY 120
CeGCP2.1b      HKSDAGVIQDNIKTENIKKYLRIFTKDPHVAGTEANKKVAYEIANAWSEAGLEDVHTLPY 101
CeGCP2.1c      HKSDAGVIQDNIKTENIKKYLRIFTKDPHVAGTEANKKVAYEIANAWSEAGLEDVHTLPY 101
               ************************************************************

CeGCP2.1a      EVLLSYPDFENPNSVIIKSSAGKEVFKSKGVSPVIIPDEQSGKYAGHQWLAYAGNGSASA 180
CeGCP2.1b      EVLLSYPDFENPNSVIIKSSAGKEVFKSKGVSPVIIPDEQSGKYAGHQWLAYAGNGSASA 161
CeGCP2.1c      EVLLSYPDFENPNSVIIKSSAGKEVFKSKGVSPVIIPDEQSGKYAGHQWLAYAGNGSASA 161
               ************************************************************

CeGCP2.1a      DVVYINHGTANDFKNLKLMGVDIKGKIALMRYGHGFRGDKIHKAQQAGAIGAILFSDTQD 240
CeGCP2.1b      DVVYINHGTANDFKNLKLMGVDIKGKIALMRYGHGFRGDKIHKAQQAGAIGAILFSDTQD 221
CeGCP2.1c      DVVYINHGTANDFKNLKLMGVDIKGKIALMRYGHGFRGDKIHKAQQAGAIGAILFSDTQD 221
               ************************************************************

CeGCP2.1a      VAQDGVESENVYPKKIWMPNEGVQRGSLMHGDGDALSPYYPSKKELFKGRTIEEAKEDGV 300
CeGCP2.1b      VAQDGVESENVYPKKIWMPNEGVQRGSLMHGDGDALSPYYPSKKELFKGRTIEEAKEDGV 281
CeGCP2.1c      VAQDGVESENVYPKKIWMPNEGVQRGSLMHGDGDALSPYYPSKKELFKGRTIEEAKEDGV 281
               ************************************************************

CeGCP2.1a      LPSIPVLPVSYTTGYEILKRLSGRPAPSDWQGFVGGNLTYKLGPGFVNGEKLSINVHSEL 360
CeGCP2.1b      LPSIPVLPVSYTTGYEILKRLSGRPAPSDWQGFVGGNLTYKLGPGFVNGEKLSINVHSEL 341
CeGCP2.1c      LPSIPVLPVSYTTGYEILKRLSGRPAPSDWQGFVGGNLTYKLGPGFVNGEKLSINVHSEL 341
               ************************************************************

CeGCP2.1a      RTKRIRNVIGYIRGSEEPDSYIMLGNHFDAWVYGSIDPNSGTAVLAEVARAMMQTINETS 420
CeGCP2.1b      RTKRIRNVIGYIRGSEEPDSYIMLGNHFDAWVYGSIDPNSGTAVLAEVARAMMQTINETS 401
CeGCP2.1c      RTKRIRNVIGYIRGSEEPDSYIMLGNHFDAWVYGSIDPNSGTAVLAEVARAMMQTINETS 401
               ************************************************************

CeGCP2.1a      WKPARTIVFNAWDAEEFGLIGSTEFVEEFVNILQKRAVVYINMDCIQGNISLHVDTVPIL 480
CeGCP2.1b      WKPARTIVFNAWDAEEFGLIGSTEFVEEFVNILQKRAVVYINMDCIQGNISLHVDTVPIL 461
CeGCP2.1c      WKPARTIVFNAWDAEEFGLIGSTEFVEEFVNILQKRAVVYINMDCIQGNISLHVDTVPIL 461
               ************************************************************

CeGCP2.1a      EHAVIEASKQVENPSKRERSRGRKTLYDTWMKVFPDKKAGVPKIRVPGGGSDHAPFLNFA 540
CeGCP2.1b      EHAVIEASKQVENPSKRERSRGRKTLYDTWMKVFPDKKAGVPKIRVPGGGSDHAPFLNFA 521
CeGCP2.1c      EHAVIEASKQVENPSKRERSRGRKTLYDTWMKVFPDKKAGVPKIRVPGGGSDHAPFLNFA 521
               ************************************************************

CeGCP2.1a      GVPVINFTFKNYTTWDTYPLYHTMYETPFSNIHLLDTDNLSVHKAIGQYWAELAKTFADD 600
CeGCP2.1b      GVPVINFTFKNYTTWDTYPLYHTMYETPFSNIHLLDTDNLSVHKAIGQYWAELAKTFADD 581
CeGCP2.1c      GVPVINFTFKNYTTWDTYPLYHTMYETPFSNIHLLDTDNLSVHKAIGQYWAELAK----- 576
               *******************************************************

CeGCP2.1a      VILPMNTTHFASVMLKTYLPQLKTTISGINVSRSDFEDIRTQYALLSKSAQDLLTMSKKF 660
CeGCP2.1b      VILPMNTTHFASVMLKTYLPQLKTTISGINVSRSDFEDIRTQYALLSKSAQDLLTMSKKF 641
CeGCP2.1c      ------------------------------------------------------------ 576


CeGCP2.1a      QETIHFTQHSFSQNPYDPKHVNAVNERLKSTERCFINPRGVSMHNPSARHVLFSVSDSDS 720
CeGCP2.1b      QETIHFTQHSFSQNPYDPKHVNAVNERLKSTERCFINPRGVSMHNPSARHVLFSVSDSDS 701
CeGCP2.1c      ------------------------------------------------------------ 576


CeGCP2.1a      YSSSLMAGVQNAINSYDLNPTKKGLREIINQISIVQYSVICVVNTLRDVI   770
CeGCP2.1b      YSSSLMAGVQNAINSYDLNPTKKGLREIINQISIVQYSVICVVNTLRDVI   751
CeGCP2.1c      --------------------------------------------------   576
```

Figure S3. Primary sequence alignment of three *C. elegans* GCP2.1 splice variants. Performed using the Clustal Omega multiple protein alignment tool (1). Sequences were downloaded from the WormBase database (2) (WS285 version of WormBase) – GCP-2.1,

isoform a (WormBase ID : R57.1a), GCP-2.1, isoform b (WormBase ID : R57.1b), and GCP-2.1, isoform c (WormBase ID : R57.1c).

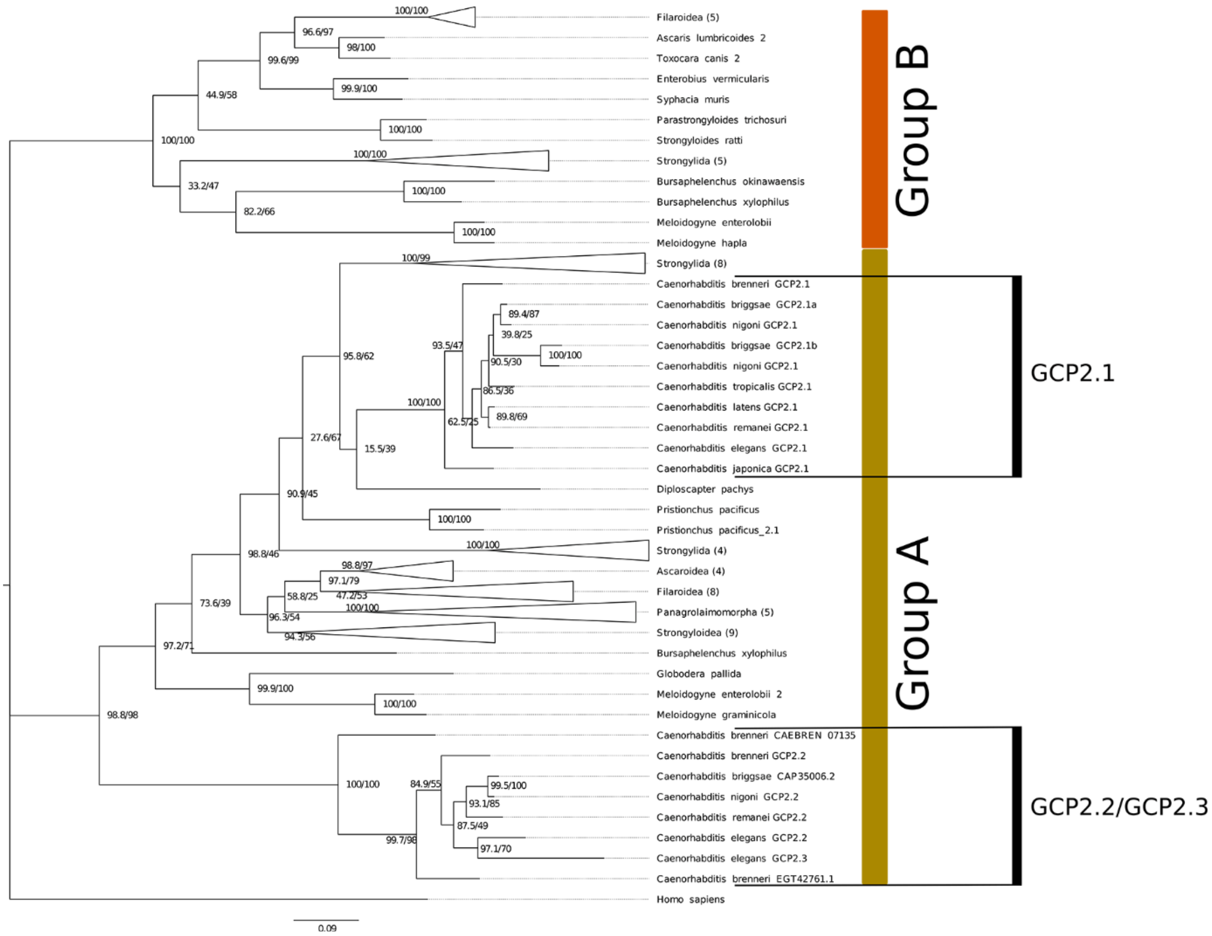

Figure S4. Maximum likelihood phylogeny tree showing the relationship between M28B peptidases of nematodes.

In the phylogeny tree *gcp-2.2* and *gcp-2.3* form a well-supported clade with their homologs from the genus *Caenorhabditis* showing that those genes evolved separately from *gcp-2.1*. The tree was constructed in IQ-TREE v 1.6.1 according to the best-fitting model (LG4M) and rooted by *Homo sapiens* GCP2. Numbers on nodes represent standard bootstrap and SH-aLRT support.

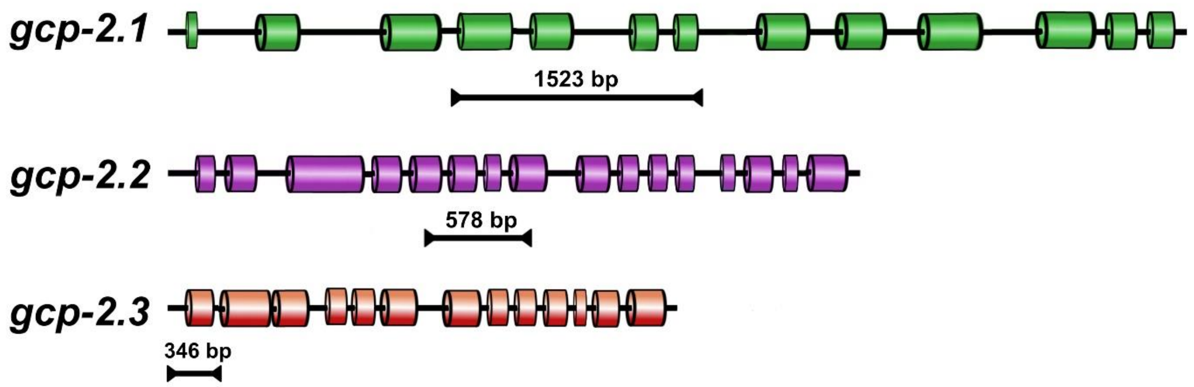

Figure S5. Scheme showing the deletion used for particular mutant worms. Horizontal bars indicate the site and length of deletion.

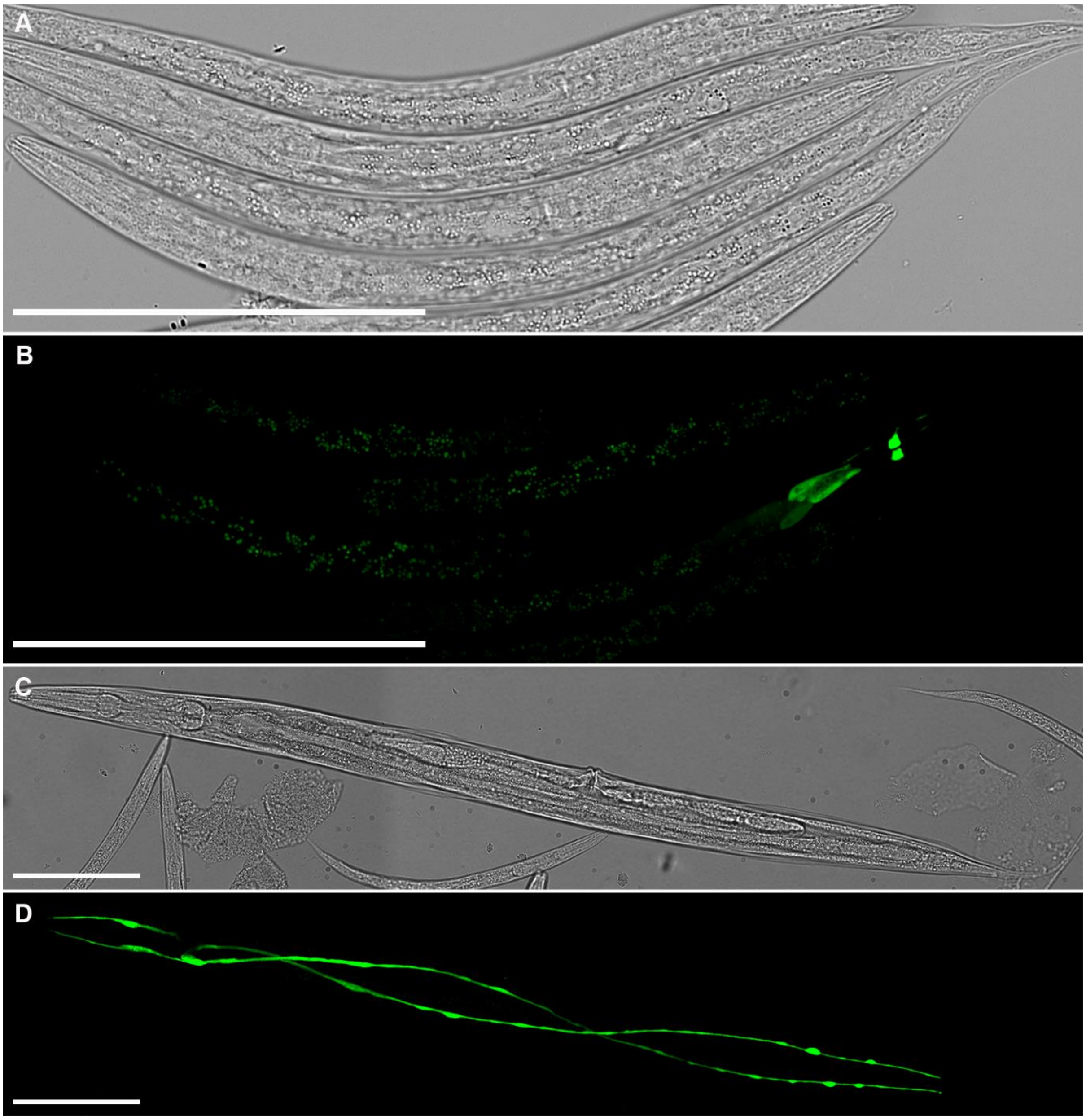

Figure S6. Localization of *gcp-2.2p::GFP* expression in the phasmid neurons (PHA and PHB) and their processes of L3 larvae.

A – Bright field image of *C. elegans* L3 larvae.

B - Same as for *gcp-2.2* in adults, the GFP signal corresponding to *gcp-2.2* expression was exclusively observed in phasmids neurons (PHAL/R and PHBL/R) and their processes in the tail of *C. elegans* larvae. One larva carrying GFP reporter construct among the negative larvae. The mosaicism is due to the extrachromosomal expression of the transgene.

C, D - Expression of *gcp-2.3p::GFP* in the excretory tissue of L4 larvae of *C. elegans*.

C – Bright field of L4 larvae.

D - The localization of *gcp-2.3p::GFP* expression was the same as in adults, and it was exclusively located in the excretory canal cell (H-shape cell and its processes).

The scale bars represent 100 µm.

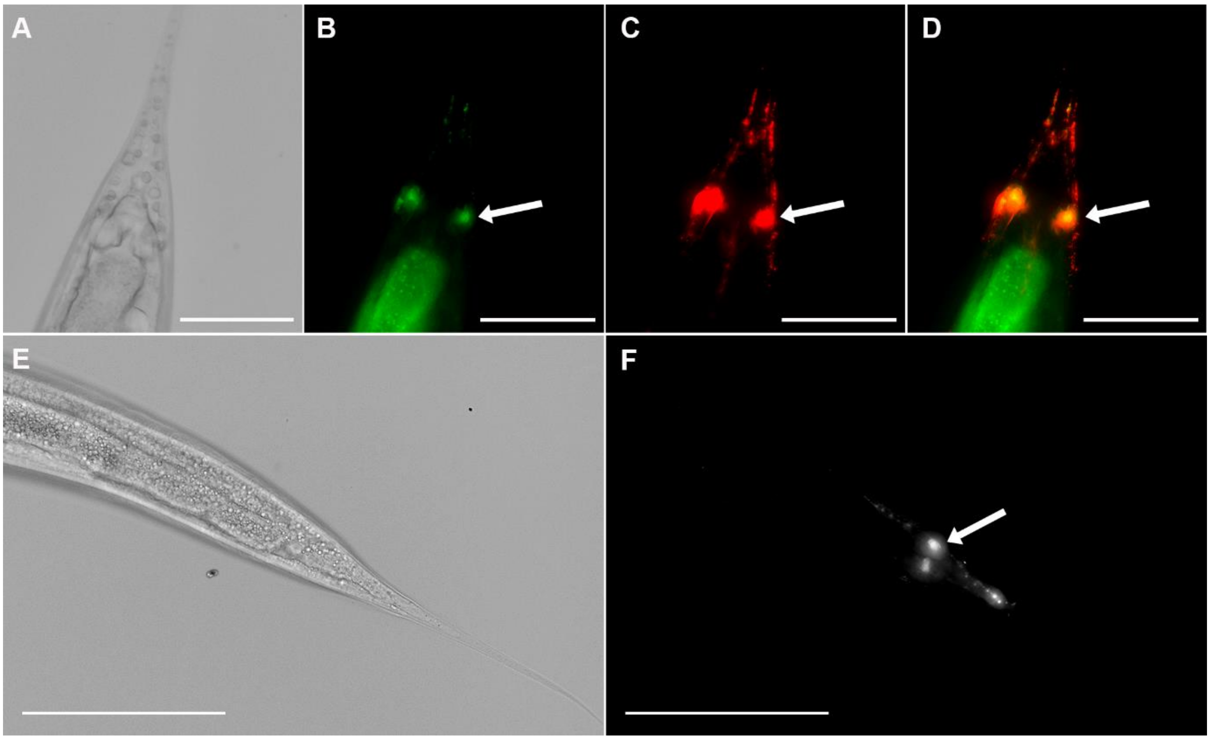

Figure S7. Identification of a pair of phasmid neurons PHA and PHB in a transgenic *C. elegans* worm.

A - DIC image of *C. elegans* tail.

B - Localization of *gcp-2.2p::GFP* expression.

C - DiI stained phasmid neurons (PHAL/R, PHBL/R).

D - Merged images allow to see that *gcp-2.2p::GFP* expression corresponds to DiI filling phasmid neurons PHA and PHB.

Arrows indicate phasmid neurons. Scale bars represent 50 µm.

E - DIC image of *C. elegans* tail.

F - Fluorescent visualization (DiI signal) of phasmid sensory neurons (arrow) and their processes in the tail of *C. elegans gcp-2.2* KO L4 larvae. Scale bars represent 100 µm.

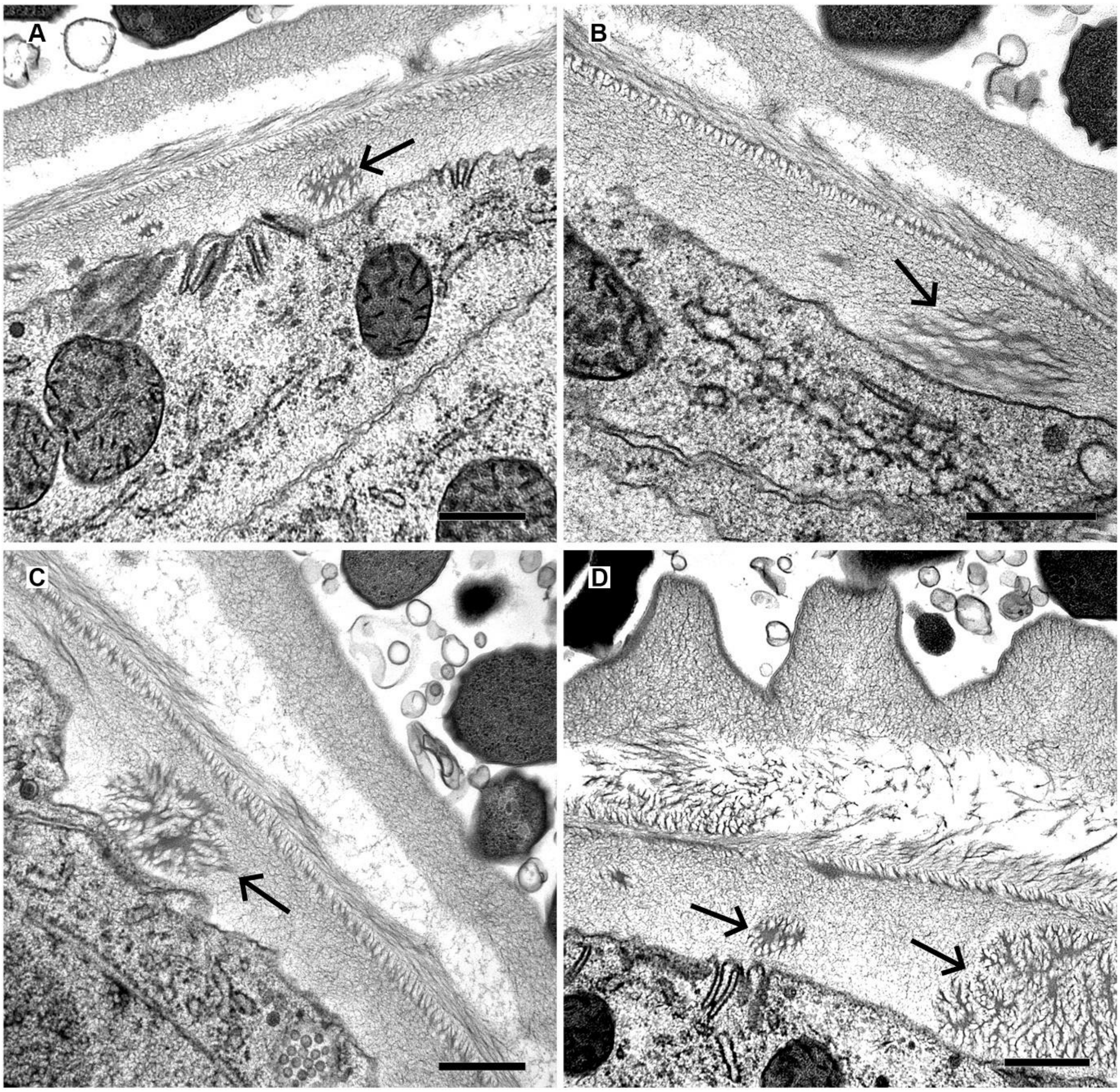

Figure S8. The cuticle structure of *gcp-2.2* knockout strain of *C. elegans*.
Several views (A-D) on unspecific spongy structures situated in the basal layer of the cuticle (arrows). Scale bars show 500 nm.

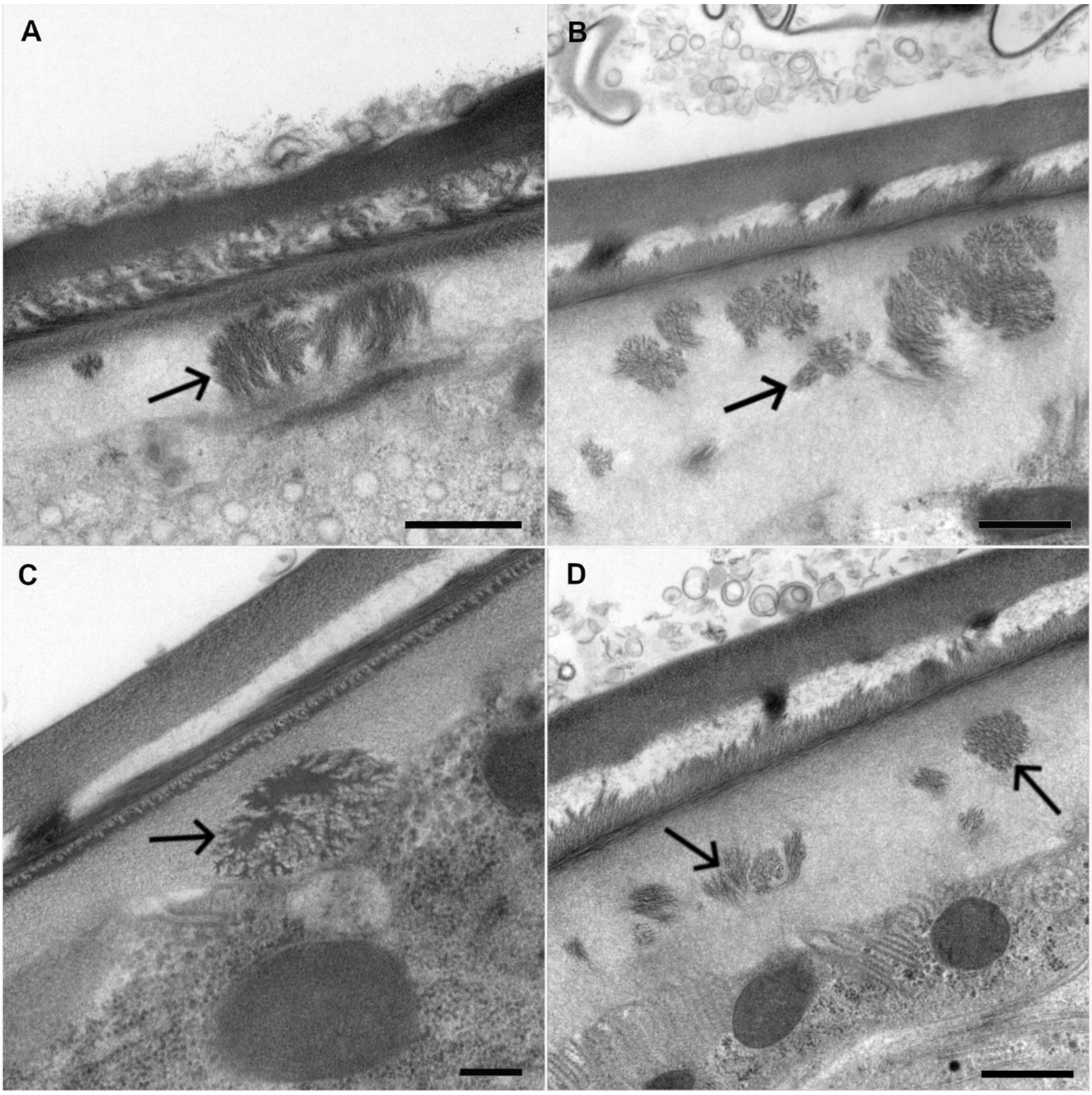

Figure S9. The cuticle structure of *gcp-2.2 gcp-2.3* double mutant *C. elegans* worms.
To corroborate our prior ultrastructural observations regarding *gcp-2.2* mutant worms, we conducted an examination of double mutant worms featuring knockouts for both *gcp-2.2* and *gcp-2.3*. Additionally, this mutation included the deletion of neighboring genes, specifically C35C5.8, C35C5.9, C35C5.3, C35C5.4, and C35C5.14. However, we supposed that the ultrastructural alterations in the basal cuticle layer are a consequence of the *gcp-2.2* knockout, as the structural changes in the basal layer closely resemble those seen in *gcp-2.2* mutant worms.
Multiple perspectives on unspecific spongy formations located within the basal layer of the cuticle (indicated by arrows). **A**, **B**, **D**, - scale bars show 500 nm; **C** – scale bar shows 200 nm.

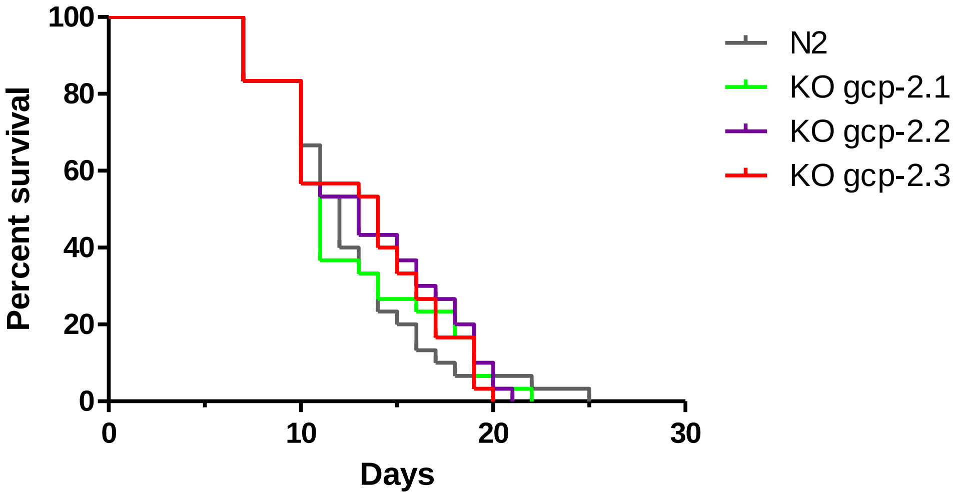

Figure S10. The effect of the gene knockout on the lifespan of *C. elegans* worms.

Table S1. *C. elegans* strains used in the study.

Gene	Strain name	Genotype/Allele Name (WormBase)	Sequence Name	Source
	N2	*C. elegans* wild isolate		CGC[1]
gcp-2.1	RB1055	ok1004	R57.1	CGC[1]
gcp-2.2	Gcp-2.2	tm6541	C35C5.2	Dr. Mitani/NBRP[2]
gcp-2.3	C35C5.11	tm5414	C35C5.11	Dr. Mitani/NBRP[2]

[1] *Caenorhabditis* Genetics Center, University of Minnesota, MN, (Ann E. Rougvie)
[2] National Bioresource Project for the Experimental Animal "Nematode *C. elegans*"

Table S2. Primers designed for genotyping after backcrossing and for amplification of promoter regions of *gcp-2.1*, *gcp-2.2* and *gcp-2.3* (restriction sites underlined).

Primers for genotyping after backcrossing

gcp-2.1	
R57_lex3-F	AAATGCGTGGTCGGAAGCAG
R57_1ex5-R	TCTGAACACCCTCATTAGGC
R57_1ex8-R	GATCCATAGACCCATGCGTC
gcp-2.2	
genGCP2.2IntFwd	ACGTTGACAACATTCGCTCC
genGCP2.2IntRev	ACATCGCCTGAGTTTCTAGT
gcp-2.3	
genGCP2.3ExtFwd	CAACGGAAGTTTGATGCCTG
genGCP2.3ExtRev	GAAGGGAATGCCGGCAATCT

Primers for amplification of promotor regions

gcp-2.1	
R57proF	AAAAAAGCTTCTCAACCACTTCATTTATTTATC
R57proR	AAAAGGATCCTTACATATAAACTATTGTTCTGGAAC
	restriction sites – HindIII and BamHI
gcp-2.2	
FRD2.2promBamHI	GATAGGATCCAGAGATAATTTTCATTTCTATGATTGTCC
REV2.2promKpnI	GTAAGGTACCTTTCTACACTTAAATTAGGATCAAATTTTAC
	restriction sites – BamHI and KpnI
gcp-2.3	
FRD2.3promBamHI	GATAGGATCCTCACGTTTGATAGACTCTCAGAGTAA
REV2.3promKpnI	GTAAGGTACCGTTCGATTTTTGAAAAACGTTATATC
	restriction sites – BamHI and KpnI

Table S3. PCR protocol for analyses of homozygous or heterozygous lines of KO worms after backcrossing.

KO *gcp-2.1*
PCR mix:

Ready To Use PCR MasterMix 12	12.5 µl
R57_1ex3-F	0.5 µl
R57_1ex5-R	0.5 µl
R57_1ex8-R	0.5 µl
DNA template	1 µl
PCR-grade water	10 µl

cycler setting:

initial denaturation	3 min	94 °C	1x
denaturation	30 sec	94 °C	40x
annealing	1 min	53 °C	40x
extension	3 min	72 °C	40x
final extension	5 min	72 °C	1x

KO *gcp-2.2*
PCR mix:

Ready To Use PCR MasterMix 12	12.5 µl
genGCP2.2IntFwd	0.5 µl
genGCP2.2IntRev	0.5 µl
DNA template	1 µl
PCR-grade water	10.5 µl

cycler setting:

initial denaturation	3 min	94 °C	1x
denaturation	30 sec	94 °C	35x
annealing	1 min	53 °C	35x
extension	2 min	72 °C	35x
final extension	5 min	72 °C	1x

KO *gcp-2.3*
PCR mix:

Ready To Use PCR MasterMix 12	12.5 µl
genGCP2.3ExtFwd	0.5 µl
genGCP2.3ExtRev	0.5 µl
DNA template	0.5 µl
PCR-grade water	11 µl

cycler setting:

initial denaturation	3 min	94 °C	1x
denaturation	30 sec	94 °C	35x
annealing	1 min	58.5 °C	35x
extension	2 min	72 °C	35x
final extension	5 min	72 °C	1x

Table S4. Statistical evaluation of the data for the impact of gene knockouts on the reproduction of the worm (Fig. 7 A in the article).

**Kruskal-Wallis test**	
P value	0.0054
Exact or approximate P value?	Gaussian Approximation
P value summary	**
Do the medians vary signif. (P < 0.05)	Yes
Number of groups	4
Kruskal-Wallis statistic	12.69

**Dunn's Multiple Comparison Test Summary**	**Difference in rank sum**	**Significant? P < 0.05?**	**Summary**
N2 vs KO gcp-2.1	7.600	No	ns
N2 vs KO gcp-2.2	12.80	Yes	**
N2 vs KO gcp-2.3	9.600	No	ns
KO gcp-2.1 vs KO gcp-2.2	5.200	No	ns
KO gcp-2.1 vs KO gcp-2.3	2.000	No	ns
KO gcp-2.2 vs KO gcp-2.3	-3.200	No	ns

Explanation of characters used in the tables. P value < 0.001 - Extremely significant (***); P value 0.001 to 0.01 - Very significant (**); P value 0.01 to 0.05 – Significant (*);
P value > 0.05 - Not significant (ns).

Table S5. Statistical evaluation of the data for the impact of gene knockouts on pharyngeal pumping (Fig. 7 B in the article).

**Kruskal-Wallis test**	
P value	< 0.0001
Exact or approximate P value?	Gaussian Approximation
P value summary	****
Do the medians vary signif. (P < 0.05)	Yes
Number of groups	4
Kruskal-Wallis statistic	30.12

**Dunn's Multiple Comparison Test Summary**	**Difference in rank sum**	**Significant? P < 0.05?**	**Summary**
N2 vs KO gcp-2.1	-22.60	Yes	***
N2 vs KO gcp-2.2	-12.15	No	ns
N2 vs KO gcp-2.3	2.750	No	ns
KO gcp-2.1 vs KO gcp-2.2	10.45	No	ns
KO gcp-2.1 vs KO gcp-2.3	25.35	Yes	***
KO gcp-2.2 vs KO gcp-2.3	14.90	Yes	*

Explanation of characters used in the tables. P value < 0.001 - Extremely significant (***); P value 0.001 to 0.01 - Very significant (**); P value 0.01 to 0.05 – Significant (*); P value > 0.05 - Not significant (ns).

Table S6. Statistical evaluation of the data for the impact of gene knockouts on resistance to hyperosmotic stress (Fig. 9 in the article).

(a) Effect of 300 mM NaCl on the duration of life span of *C. elegans* mutants.

**Kruskal-Wallis test**	
P value	< 0.0001
Exact or approximate P value?	Gaussian Approximation
P value summary	****
Do the medians vary signif. (P < 0.05)	Yes
Number of groups	4
Kruskal-Wallis statistic	21.22

**Dunn's Multiple Comparison Test**	**Difference in rank sum**	**Significant? P < 0.05?**	**Summary**
N2 vs KO gcp-2.1	17.68	No	ns
N2 vs KO gcp-2.2	2.475	No	ns
N2 vs KO gcp-2.3	-15.25	No	ns
KO gcp-2.1 vs KO gcp-2.2	-15.20	No	ns
KO gcp-2.1 vs KO gcp-2.3	-32.93	Yes	***
KO gcp-2.2 vs KO gcp-2.3	-17.73	No	ns

Explanation of characters used in the tables. P value < 0.001 - Extremely significant (***); P value 0.001 to 0.01 - Very significant (**); P value 0.01 to 0.05 – Significant (*);
P value > 0.05 - Not significant (ns).

(b) Effect of 400 mM NaCl on the duration of life span of *C. elegans* mutants.

**Kruskal-Wallis test**	
P value	0.0012
Exact or approximate P value?	Gaussian Approximation
P value summary	**
Do the medians vary signif. (P < 0.05)	Yes
Number of groups	4
Kruskal-Wallis statistic	15.84

**Dunn's Multiple Comparison Test**	**Difference in rank sum**	**Significant? P < 0.05?**	**Summary**
N2 vs KO gcp-2.1	25.28	Yes	**
N2 vs KO gcp-2.2	17.00	No	ns
N2 vs KO gcp-2.3	5.125	No	ns
KO gcp-2.1 vs KO gcp-2.2	-8.275	No	ns
KO gcp-2.1 vs KO gcp-2.3	-20.15	Yes	*
KO gcp-2.2 vs KO gcp-2.3	-11.88	No	ns

Explanation of characters used in the tables. P value < 0.001 - Extremely significant (***); P value 0.001 to 0.01 - Very significant (**); P value 0.01 to 0.05 – Significant (*);
P value > 0.05 - Not significant (ns).
